# Identification of key ferroptosis-related biomarkers in steroid-induced osteonecrosis of the femoral head based on machine learning

**DOI:** 10.1186/s13018-023-03800-x

**Published:** 2023-04-29

**Authors:** Jian Liu, Xueliang Han, Lianjun Qu, Bencai Du

**Affiliations:** Department of Orthopedic, Sunshine Union Hospital, 9000 Yingqian Road, High-Tech Zone, Weifang, 261000 Shandong China

**Keywords:** Steroid-induced osteonecrosis of the femoral head, Ferroptosis, Immune, Machine learning

## Abstract

**Background:**

This study was aimed to identify key ferroptosis-related biomarkers in steroid-induced osteonecrosis of the femoral head (SONFH) based on machine learning algorithm.

**Methods:**

The SONFH dataset GSE123568 (including 30 SONFH patients and 10 controls) was used in this study. The differentially expressed genes (DEGs) were selected between SONFH and control groups, which were subjected to WGCNA. Ferroptosis-related genes were downloaded from FerrDb V2, which were then compared with DEGs and module genes. Two machine learning algorithms were utilized to identify key ferroptosis-related genes, and the underlying mechanisms were analyzed by GSEA. Correlation analysis between key ferroptosis-related genes and immune cells was analyzed by Spearman method. The drug–gene relationships were predicted in CTD.

**Results:**

Total 2030 DEGs were obtained. WGCNA identified two key modules and obtained 1561 module genes. Finally, 43 intersection genes were identified as disease-related ferroptosis-related genes. After LASSO regression and RFE-SVM algorithms, 4 intersection genes (AKT1S1, BACH1, MGST1 and SETD1B) were considered as key ferroptosis-related gene. The 4 genes were correlated with osteoclast differentiation pathway. Twenty immune cells with significant differences were obtained between the groups, and the 4 key ferroptosis-related genes were correlated with most immune cells. In CTD, 41 drug–gene relationship pairs were finally obtained.

**Conclusions:**

The 4 key ferroptosis-related genes, *AKT1S1*, *BACH1*, *MGST1* and *SETD1B*, were identified to play a critical role in SONFH progression through osteoclast differentiation and immunologic mechanisms. Additionally, all the 4 genes had good disease prediction effect and could act as biomarkers for the diagnosis and treatment of SONFH.

**Supplementary Information:**

The online version contains supplementary material available at 10.1186/s13018-023-03800-x.

## Background

Steroid-induced osteonecrosis of the femoral head (SONFH) is a devastating disease that usually progresses to osteoarthritis of the hip joint and femoral head collapse [1]. The most common symptoms of SONFH are movement limitation and severe pain [2]. The male gender and longer symptom duration are the risk factor for poor prognosis [3]. The current treatment measures for osteonecrosis of the femoral head (ONFH) mainly included operative management [4] and conservative treatment, such as osteotomy [5], core decompression [6] and bone marrow-derived cell therapies [7]. Given that the outcomes of ONFH were heterogeneous, there is no most approved therapy for ONFH patients. In China, there are around 1.5 × 10^5^–2 × 10^5^ new SONFH cases annually [8]. Thus, it is imperative to discover the novel biomarkers for the diagnosis and treatment of SONFH. Although the etiology and pathogenesis of SONFH have been extensively studied [9], there is no clear consensus on its exact origin.

Ferroptosis is a recently discovered form of iron-mediated cell death, which causes much attention as new regulated necrosis [10]. Ferroptosis is characterized by an increased level of lipid peroxidation products and reactive oxygen species [11, 12]. The morphological characteristics of ferroptosis include the obvious shrinkage of cell mitochondria, reduction or disappearance of mitochondrial crest, but the cell membrane is intact with normal nucleus size [13]. Dysregulation of ferroptosis is correlated with a lot of pathological processes, such as inflammation-related diseases, neurodegenerative diseases, and cancers [14–16]. Recently, Sun et al. [17] reported that dexamethasone could induce ferroptosis through the pathway of P53/SLC7A11/GPX4 in glucocorticoid-induced osteonecrosis of the femoral head. Whereas, the study on the roles of ferroptosis in SONFH is still scarce.

Presently, this study was aimed to analyze key ferroptosis-related biomarkers in SONFH based on machine learning algorithm. The current findings may offer new insights on the SONFH pathogenesis, thereby providing new strategies for its diagnosis and treatment.

## Methods

### Data acquisition

The SONFH dataset GSE123568 was downloaded from NCBI Gene Expression Omnibus database, which included 30 SONFH patients and 10 non-SONFH patients as control. The GPL15207 [PrimeView] Affymetrix Human Gene Expression Array platform was used for gene-chip assays. This dataset was used as analytical dataset.

### Data preprocessing

For the above gene-chip dataset, the preprocessed, standardized and log2 transformed probe expression matrix were downloaded, and then, the annotation files were downloaded. Through one-to-one matching of probe and gene symbol, the probe that did not match gene symbol was deleted. For different probes that mapped to the same gene, the mean value was taken as the expression value of this gene for subsequent analysis.

### Differentially expressed gene analysis

Based on the analysis dataset, the classical Bayesian method in limma 3.10.3 [18] was used for differentially expressed gene (DEGs) analysis of SONFH vs. control. The p value was corrected by Benjamini & Hochberg algorithm. The differential expression thresholds were set as adj.*p*.value < 0.05 and |logFC (fold change)|< 0.5. After obtaining the DEGs, the volcano map and heatmap were drawn, respectively, for visual display.

### Screening of SONFH correlated module genes

Weighted gene co-expression network analysis (WGCNA) is an analysis method to cluster the genes with similar expression patterns and then distinguish modules by gene expression similarity. The correlations between modules and modules, as well as modules and sample traits were calculated, so as to screen modules with highly correlated traits. Additionally, the genes in the significant module could be analyzed to find the target genes related to the research.

In order to find out the module genes highly related to diseases in the dataset GSE123568, all the genes were ranked in the dataset according to the variance from largest to smallest. Then, the genes with the top 25% variance (total number:12050) were selected and the disease status of the samples was taken as traits for analysis using the R package WGCNA1.61 [19].

### SONFH-related ferroptosis-related gene (FRG) analysis

Firstly, FRGs were retrieved from FerrDb V2 [20]. After that, the intersection of DEGs, FRGs, and disease-highly associated module genes obtained from WGCNA analysis was used as SONFH-related FRGs for subsequent analysis.

### Functional enrichment analyses

Based on these disease-related FRGs, the GO (Gene Ontology) function [21] and KEGG (Kyoto Encyclopedia of Genes and Genomes) pathways [22] were analyzed using DAVID [23]. The number of enriched genes in each term was set as at least 2. A *p* < 0.05 was considered as the threshold vale. The top 10 GO entries and top 20 KEGG pathways were selected for display.

### Protein–protein interaction (PPI) network analysis

In order to understand the protein interactions between disease-related FRGs, the online database STRING 11.0 [24] was used to predict and analyze whether there was any interaction between gene-encoded proteins. PPI score was set as 0.4. Cytoscape 3.4.0 [25] was used to construct PPI networks. In addition, CytoNCA [26] plug-in 2.1.6 was used to analyze the topological properties, including “degree,” “betweenness” and “closeness of nodes” in the network.

### Screening of key FRGs

SONFH-related FRGs (number: 43) screened above were further filtered by two machine learning algorithms as previously described [27, 28].

1. The expression values of disease-related FRGs in various samples were extracted. Then, LASSO algorithm was used to screen the feature genes combined with sample grouping. The glmnet package 4.2–2 [29] of R 3.6.1 was used for regression analysis, and the parameter was set as nfold = 20.
2. The support vector machine (SVM) algorithm in R package "e1071" (version1.7–9) [30] was used to sort the disease-related FRGs. The recursive feature elimination (RFE) method was used to select the best gene from GSE123568 cohort to avoid the overfitting. Briefly, the importance ranking of each gene were achieved, and the error rate and accuracy of each iteration combination were obtained. The lowest error rate was selected as the best combination, and the corresponding gene was taken as the feature gene.

Finally, the overlapped feature genes screened from LASSO regression algorithm and RFE-SVM algorithm were obtained as the key FRGs.

### Evaluation of diagnostic efficacy of key FRGs

For the key FRGs obtained from the above analyses, the expression values of the key genes in the data set were extracted, and then, the diagnostic ROC curves of the key genes were drawn by combining the sample grouping.

### Gene set enrichment analysis (GSEA)

In dataset GSE123568, Pearson correlation coefficients between each key FRG and all the other genes were calculated, respectively, and then, the correlation coefficients were sorted from largest to smallest. After obtaining the related gene set for each FRG, GSEA based of KEGG pathway enrichment was conducted by using R clusterProfiler 3.8.1 [31]. Meanwhile, Benjamini & Hochberg method was adopted to conduct multiple inspection correction, and adj.*p* < 0.05 was regarded as threshold. The top 5 KEGG pathways according to the ranking of significance were displayed.

### Correlation analysis of key FRGs and immunity

Firstly, 28 kinds of immune cells and their corresponding marker genes were obtained from a previous literature [32]. Further, based on the gene expression matrix of marker genes in all samples, ssGSEA algorithm was adopted and R package GSVA 1.36.2 [33] was used to calculate the enrichment fraction of each immune cell. Then, the immune cells with differential infiltration levels between SONFH and control group were analyzed by Wilcoxon test. The differentially infiltrating immune cells with *p* < 0.05 were considered to be closely related to SONFH.

Then, Spearman method [34] was applied to analyze the correlation between key FRGs and infiltrating immune cells, and the corresponding p value and correlation coefficient cor were obtained.

### Targeted drug prediction of key FRGs

The online database CTD [35] was used to search chemical interactions of the key genes. The drug–gene relationships supported by at least two references were selected. Cytoscape 3.4.0 was used for network building.

## Results

### Differential analysis

**Fig. 1:**
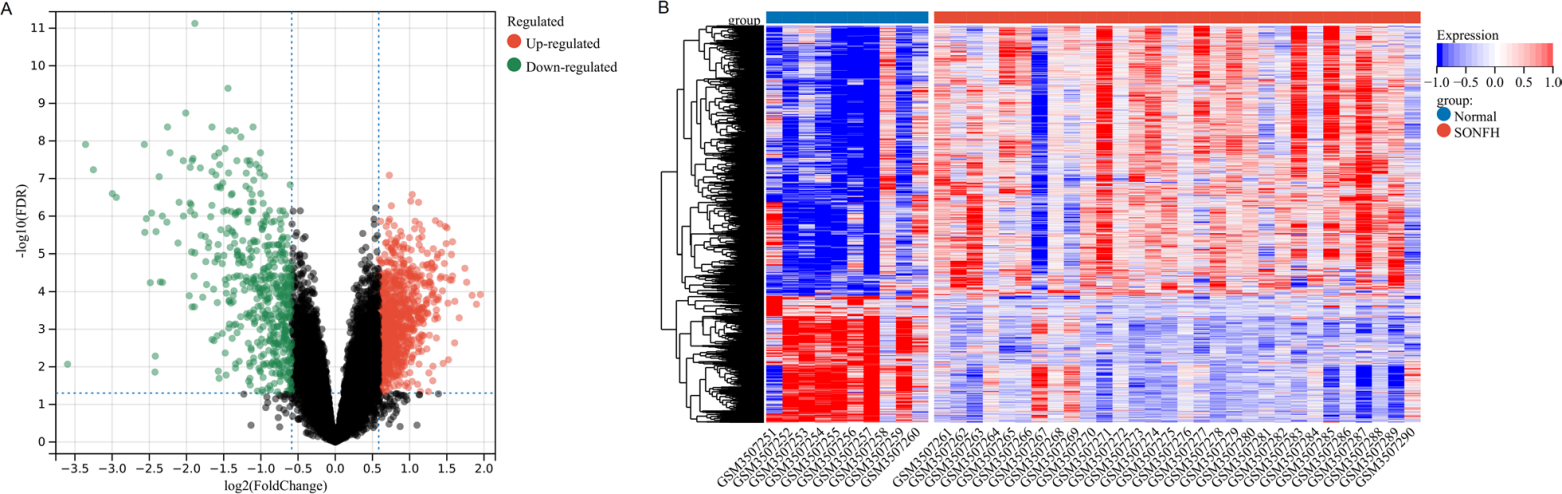
**A** Volcano plot of differentially expressed genes (green for down-regulated genes, red for up-regulated genes, black for insignificant genes). **B** Heatmap of differentially expressed genes (the top blue bars for control samples, red for disease samples)

According to the cutoff value of adj.*p*.val < 0.05 and |logFC|> 0.5, 1380 up-regulated and 650 down-regulated DEGs between SONFH and control groups were selected. The differential volcano and heatmaps are shown in Fig. 1A, B.

### Disease-related gene analysis

**Fig. 2:**
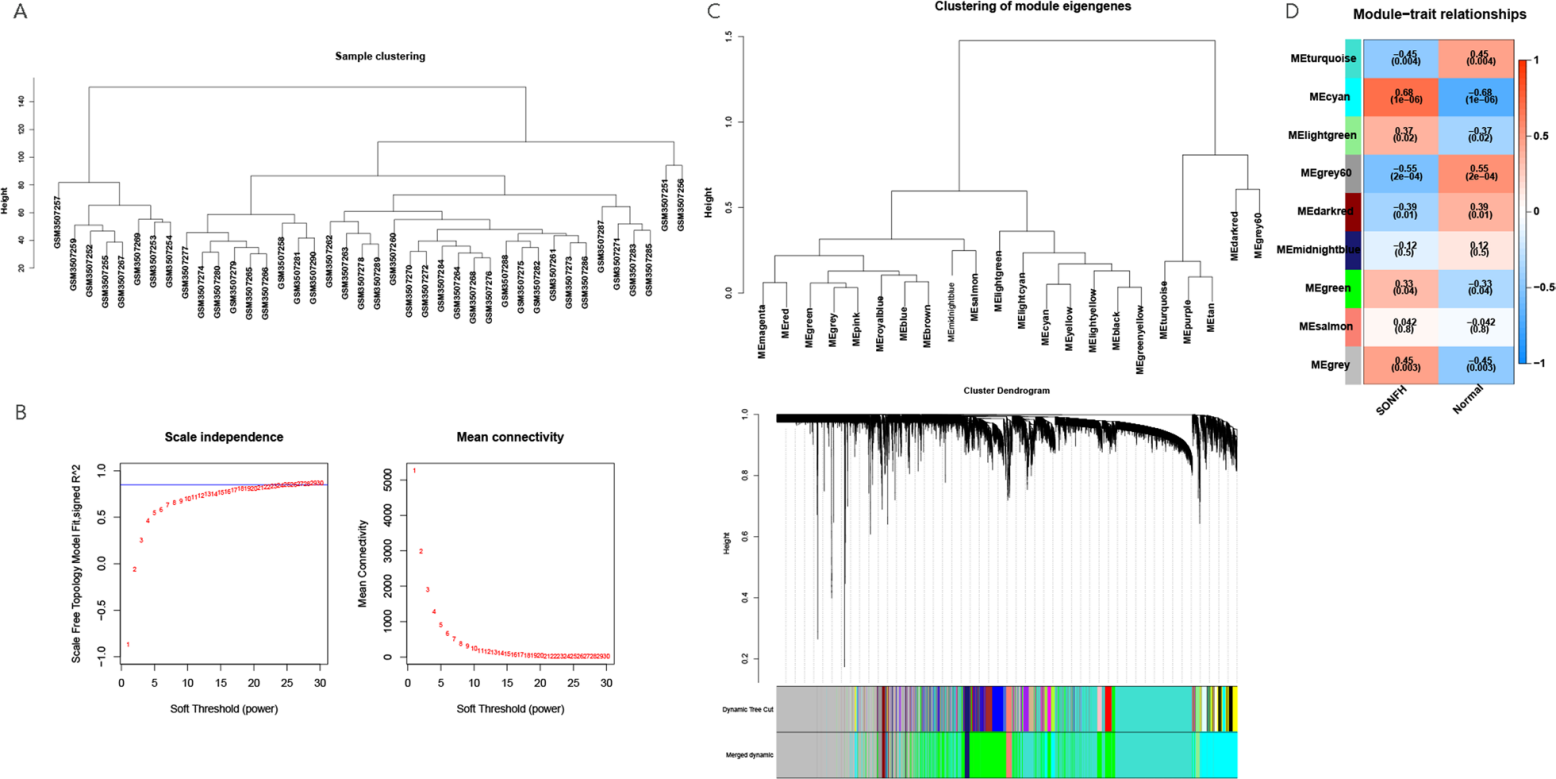
**A** Sample clustering of data set. **B** Scale-free soft threshold distribution. The vertical axis is Scale Free Topology Model Fit (signed *R*^2^). The higher the square of the correlation coefficient, the closer the network is to the scale-free distribution. The vertical axis of the right figure represents the mean value of all gene adjacency functions in the corresponding gene module. **C** Module clustering tree diagram. Genes are classified into modules by hierarchical clustering, with different colors representing different modules. **D** Correlation analysis result between WGCNA module and sample subtype. In each square, the upper number is the correlation coefficient cor and the lower number is the *p* value. The darker the color, the closer the correlation between modules with clinical traits

In order to observe the overall correlation of all samples in the dataset, the samples were clustered and the outlier samples were eliminated to ensure the accuracy of the analysis. The sample clustering based on gene expression is shown in Fig. 2A. The SONFH and control samples were clustered separately, so the samples were not eliminated. Then, in order to ensure that the interaction between genes met the scale-free distribution, we first determined the soft threshold of the data, as shown in Fig. 2B. We selected the optimal power value recommended by the WGCNA package, that was power = 25.

The adjacency among genes was analyzed, and the similarity was calculated based on the adjacency. Then, the differentiation coefficient among genes was derived, and the systematic clustering tree among genes was obtained. Then, according to the dynamic tree cutting standard, the fewest genes were set to 70 for each gene module, obtaining 22 modules. After that, MEDissThres was set to 0.2 to combine similar modules analyzed by dynamic tree algorithm. Cluster dendrogram of genes in SONFH is presented in Fig. 2C. Further, by calculating the correlation between the feature vector gene of each module and the clinical traits, 9 modules closely correlated with SONFH were obtained, of which cyan module (1425 genes) and grey60 module (136 genes) showed the strongest positive and negative correlation with the disease traits, respectively (Fig. 2D). Therefore, these two modules were regarded as the SONFH-closely related modules. The genes in the modules were considered as SONFH-related genes.

### Disease-related FRGs analysis

Based on FerrDb V2 database, total 621 FRGs were retrieved, including 264 driver, 9 markers, 238 suppressor and 110 unclassified genes. After removing the duplicate genes, 564 FRGs were obtained.

**Fig. 3:**
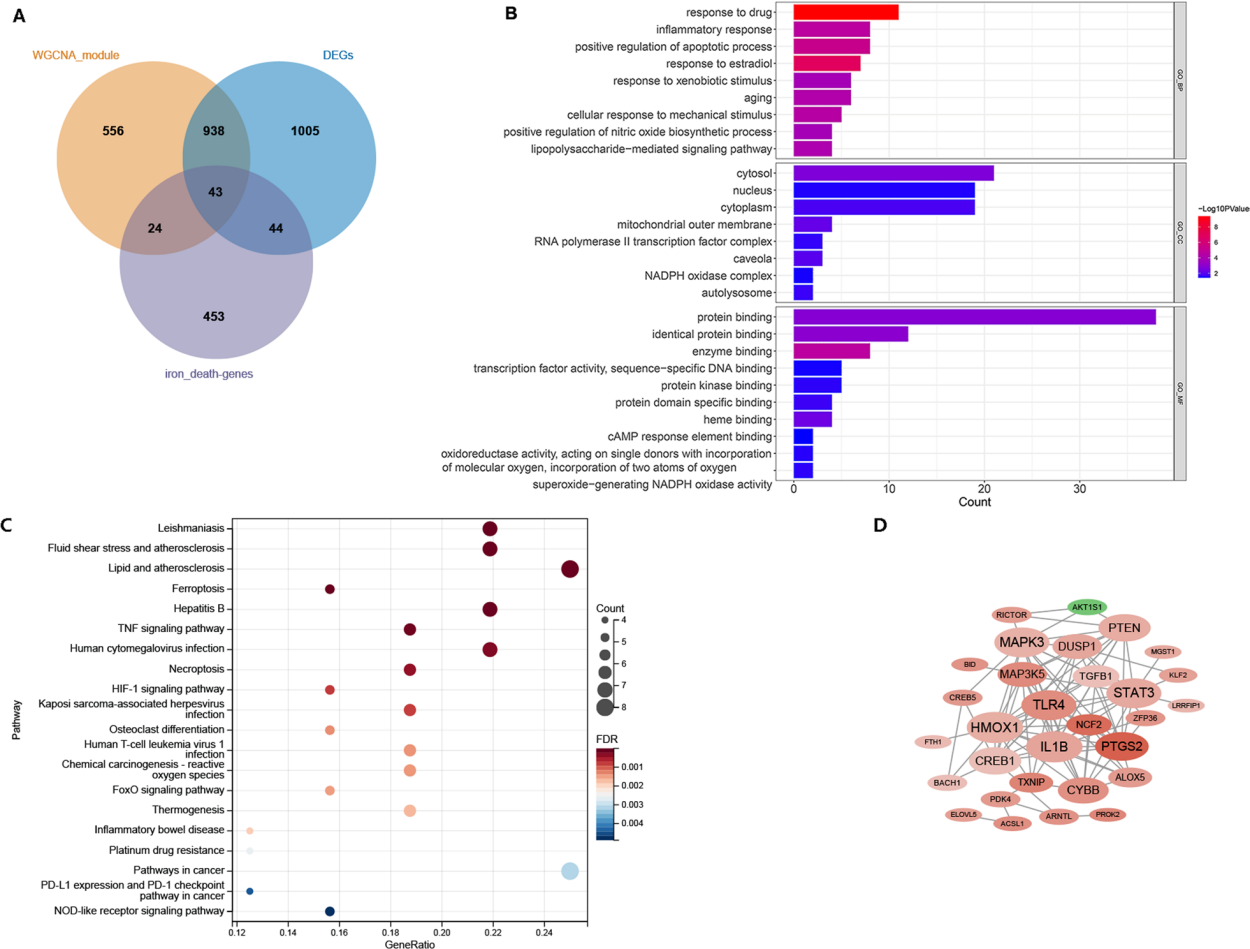
**A** Venn diagram for differentially expressed genes (DEGs), ferroptosis-related genes (FRG) and disease-related module genes (DEGs). **B** and **C**: Top10 GO (**B**) and pathways (**C**) enriched by disease-related FRGs. **D** PPI network constructed by disease-related FRG protein (red represents up-regulated gene; green represents down-regulated gene; the darker the color, the larger the multiple of difference; node size represents the degree of connectivity)

The intersection of DEGs, FRGs, and module genes obtained from WGCNA analysis was taken, and 43 genes were obtained (Fig. 3A), which were considered as disease-related FRGs for subsequent analysis.

GO and KEGG pathway analysis showed that the 43 disease-related FRGs were enriched in 84 GO-BP (biological process), 8 GO-CC (cellular component), 10 GO-MF (molecular function) terms and 52 pathways. The top 10 GO and top 20 pathways are displayed in Fig. 3B, C. The disease-related FRGs were closely related to inflammation and apoptosis-related functions, as well as TNF signaling pathway, HIF-1 signaling pathway, and osteoclast differentiation.

### Protein interaction network and correlation analysis

The protein interaction pairs of disease-related FRGs were analyzed, and 99 interaction pairs were identified, involving 30 proteins (Fig. 3D). The top ten nodes (hub nodes) were ILIB (degree, 15), HMOX1 (degree, 15), STAT3 (degree, 14), TLR4 (degree, 14), MAPK3 (degree, 14), PTGS2 (degree, 13), CREB1 (degree, 12), PTEN (degree, 12), CYBB (degree, 11), DUSP1 (degree, 10), and MAPK5 (degree, 10).

### Screening of key FRGs

**Fig. 4:**
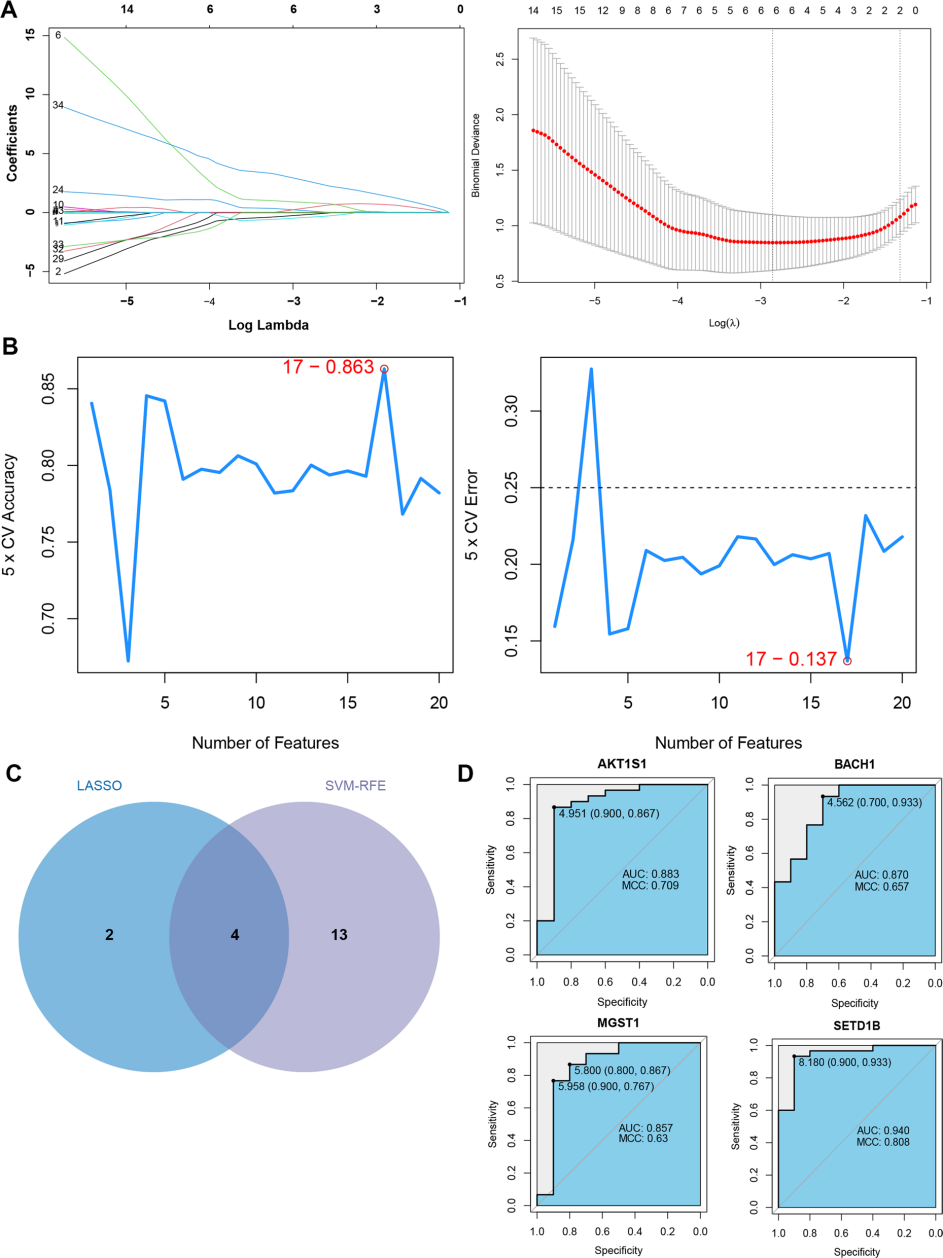
**A** LASSO regression process parameter diagram (on the left, the vertical coordinate is the coefficient of variables, and the horizontal coordinate is log (Lambda). With the change of lambda, the coefficient of most variables is finally compressed to 0; the two dotted lines in the right figure indicate two special lambda values). **B** RFE-SVM model accuracy (left) and error rate (right). Ordinate indicates the (RMSE) Root mean square error. **C** Venn diagram for the feature genes obtained by LASSO regression algorithm and RFE-SVM algorithm. **D** ROC curves of four key FRGs. MCC: Matthew’s correlation coefficient

LASSO regression analysis was conducted based on the 43 disease-related FRGs, and 6 key feature genes (AKT1S1, ARNTL, BACH1, MGST1, SETD1B and NNMT) were obtained (Fig. 4A). Additionally, RFE-SVM algorithm was also used to screen key genes, and 17 feature genes were obtained, including BACH1, AKT1S1, SETD1B, KLF2, RICTOR, MGST1, ACSL1, SAT1, LRRFIP1, PTEN, FTH1, RBMS1, PTGS2, TXNIP, TMBIM4, NCF2, and DPEP1 (Fig. 4B). Finally, the intersection of feature genes obtained by LASSO regression algorithm and RFE-SVM algorithm was taken, and 4 intersection genes (AKT1S1, BACH1, MGST1 and SETD1B) were considered as key FRGs (Fig. 4C).

According to the method, diagnostic ROC curves of the 4 key FRGs were drawn, as shown in Fig. 4D. AUC areas were all above 0.85, indicating that the 4 genes had good disease prediction effect.

### Potential mechanism of key genes explored by GSEA

**Fig. 5:**
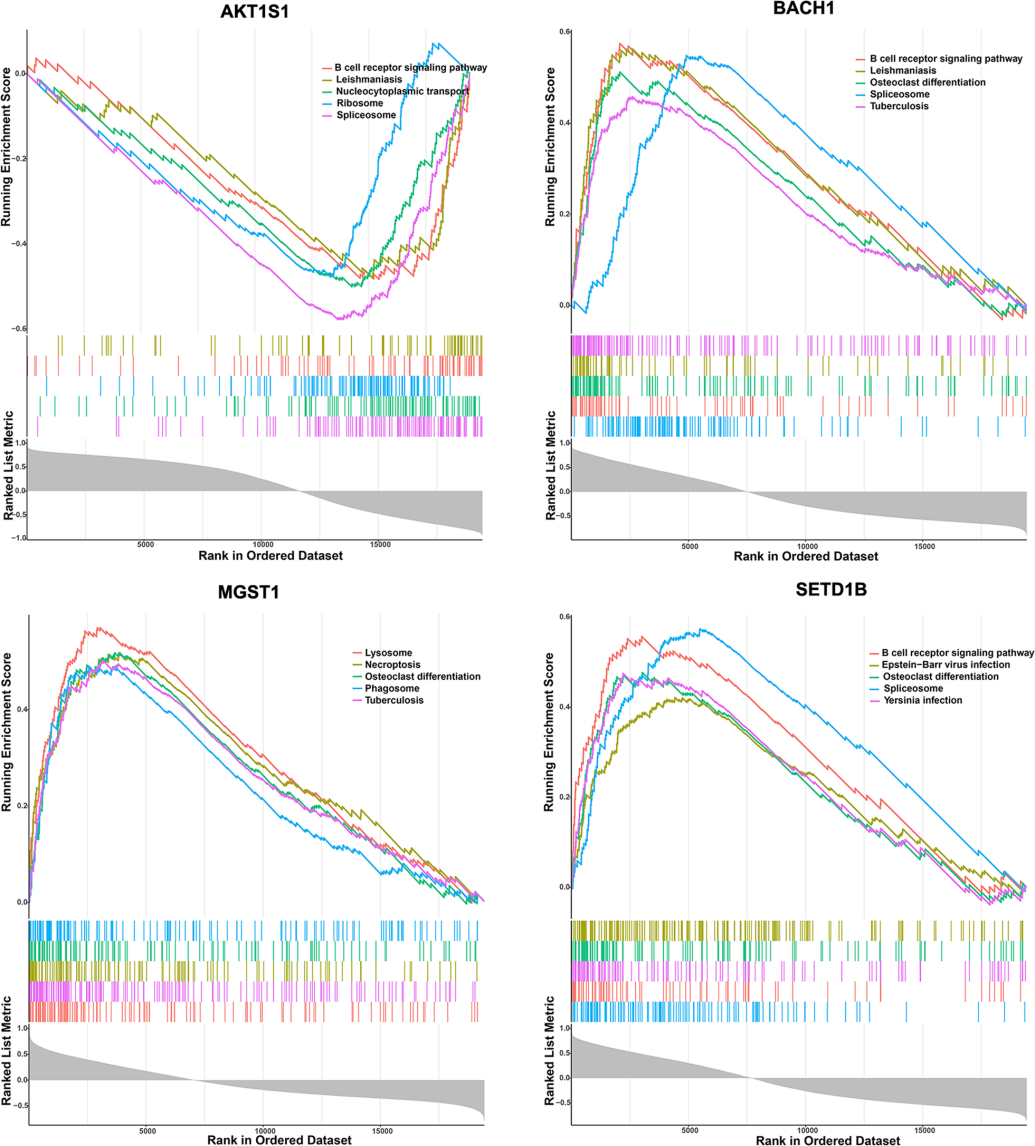
KEGG pathway analysis of 4 key FRGs by GSEA enrichment method

The KEGG pathways of 4 key FRGs were explored by GSEA. Total 47 KEGG pathways were positively associated with AKT1S1 and 118 were negatively correlated with AKT1S1; 145 positively correlated and 34 negatively correlated KEGG pathways were obtained by BACH1 enrichment; MGST1 enrichment resulted in 118 positive and 16 negative KEGG pathways; 127 positive and 28 negative KEGG pathways were obtained by SETD1B enrichment. Top 5 KEGG pathways of each gene were selected for visual display, as shown in Fig. 5. The enrichment results are detailed in Additional file 1: Tables S1–S4. The osteoclast differentiation pathway was significantly enriched, indicating that this pathway may play a key role in SONFH.

### Correlation analysis of key FRGs and immunity

**Fig. 6:**
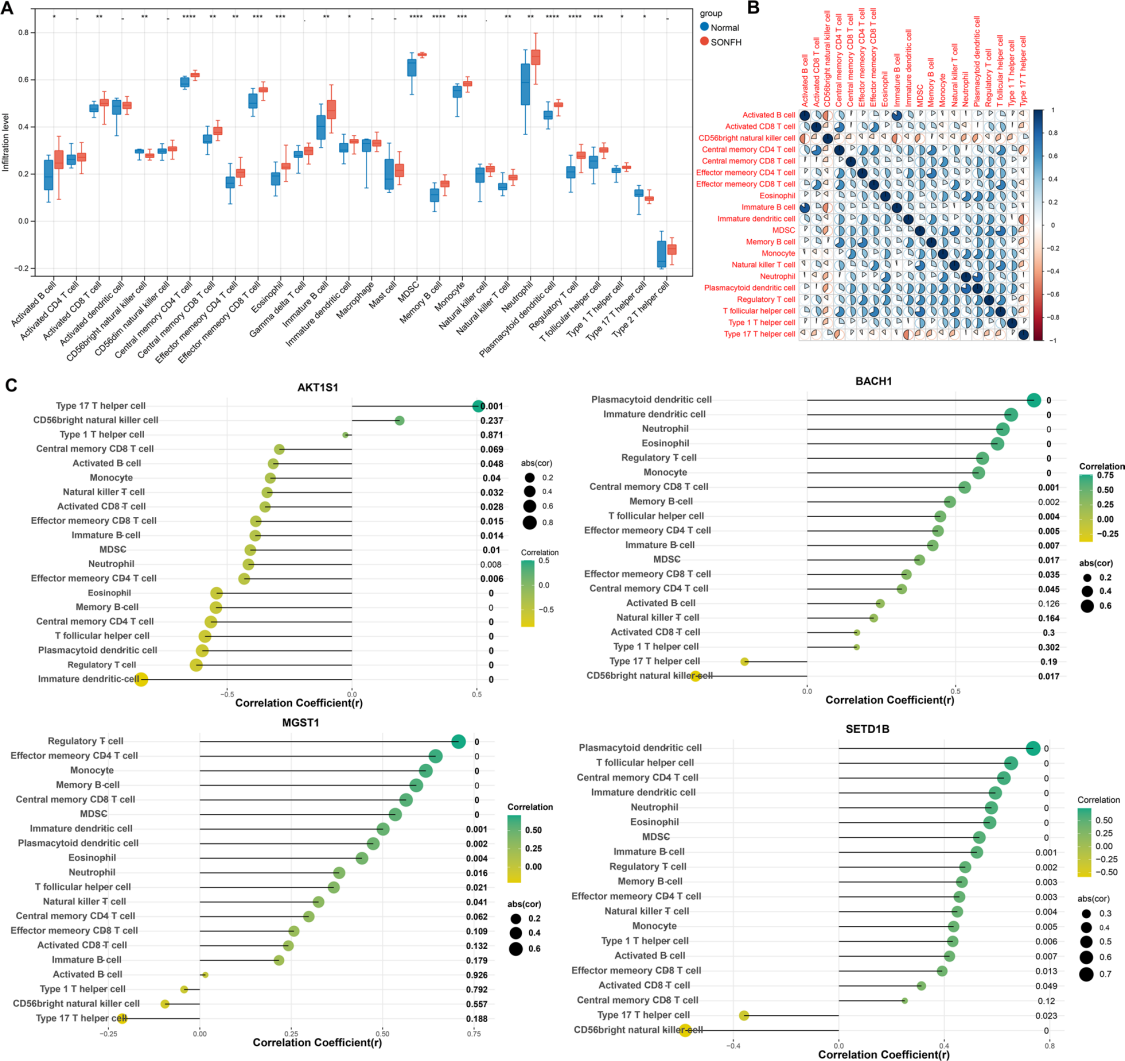
**A** The distribution of immune cell infiltration levels between disease and normal groups (*p < 0.05, ** *p* < 0.01, *** *p* < 0.001, **** *p* < 0.0001). **B** Correlation diagram of differential immune cells (red sector represents negative correlation, blue sector represents positive correlation, and larger sector area represents greater absolute value of correlation coefficient). **C** Lollipop chart of correlation between 4 key ferroptosis-related genes and 20 differential immune cells (yellow to green indicates negative to positive correlation coefficients, the larger the circle is, the greater the absolute value of correlation coefficients, and the number in the back indicates the significance of *p* value)

The enrichment scores of 28 types of immune cells were calculated to compare the differences in immune cell infiltration levels between the disease group and the normal group. As shown in Fig. 6A, there were 20 immune cells with differential infiltration levels between the groups, such as activated B cell, activated CD8 T cell, central memory CD4 T cell, eosinophil, immature dendritic cell, and neutrophil. The correlation between differential immune cells is shown in Fig. 6B. Type 17 T helper cell and CD56 bright natural killer cell were positively correlated with most types of immune cells.

In order to further understand the correlation between the screened biomarkers (4 key FRGs) and differential immune cells, the Spearman method was applied, and the corresponding p value and correlation coefficient cor were obtained. The 4 key FRGs were correlated with most immune cells (Fig. 6C).

### Targeted drug prediction

**Fig. 7:**
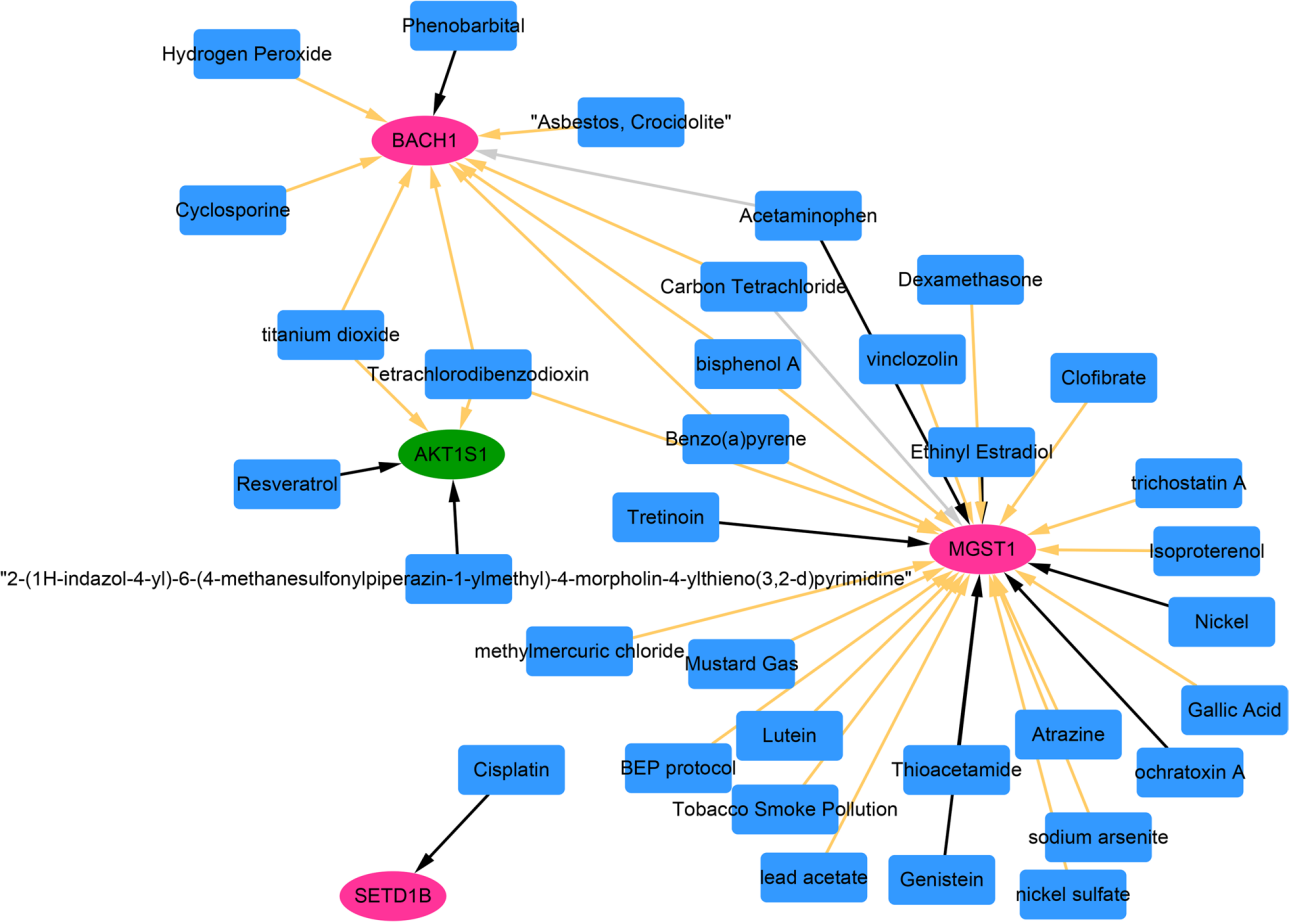
Drug–gene regulatory network (red oval represents up-regulated gene; green oval represents down-regulated gene; blue square represents small drug molecules; yellow arrow lines indicate that drugs will increase gene or protein expression; black lines indicate that drugs will reduce gene or protein expression; light gray lines indicate that drugs will affect gene or protein expression)

According to the threshold value, 41 drug–gene relationship pairs were finally obtained, including 34 drug molecules, such as phenobarbital, resveratrol, and clofibrate, and 4 key FRGs. Specially, carbon tetrachloride, bisphenol A, acetaminophen, benzo(a)pyrene, tetrachlorodibenzodioxin, and titanium dioxide had interactions with two genes (Fig. 7).

## Discussion

SONFH as a debilitating disease has become a public health burden. SONFH is usually asymptomatic; thus, the early diagnosis and prevention are imperative for SONFH patients. Emerging effects have been made for the identification of diagnostic marker for SONFH by bioinformatics approach. The study of Zhao et al. has applied WGCNA and CIBERSORT to screen the key diagnostic markers underlying the immune-related mechanism, such as TYROBP, TLR2 and P2RY13 [34]. Ferroptosis is a newly discovered type of cell death, which has been found to be implicated in various pathophysiological processes, such as infection, inflammation and immunity [36]. However, the diagnostic value of FRGs in SONFH has not clarified. Thus, in the present study, we attempted to identify the key FRGs valuable for the diagnosis and treatment for SONFH.

Our results showed that there were 4 key FRGs: *AKT1S1*, *BACH1*, *MGST1* and *SETD1B* through machine learning algorithm. They were positively correlated with the pathway of osteoclast differentiation. Additionally, 20 immune cells showed differential infiltration levels between SONFH and control groups, and the 4 key FRGs were correlated with most immune cells. Findings of this study may help to understand the pathogenesis of SONFH as well as develop novel diagnosis and treatment targets.

AKT1S1 is a substrate of Akt and a component of the mTOR complex 1 [37]. It has been reported that AKT1S1 can suppress the activity of mTOR complex 1 [38]. mTOR is a member of mTOR complex 1, and the mTOR signaling pathway can regulate many stem cell processes, including cell survival, proliferation, and differentiation [39]. Recent study revealed that the mTOR pathway may mediate the bone homeostasis [40]. Activation of the mTOR signaling pathway can impair the osteogenic/adipogenic lineage differentiation of bone marrow mesenchymal stem cells (BMSCs) [41]. Importantly, involvement of BMSCs in osteoblast lineage and bone formation is thought to be a major mediator of SONFH [42]. In this study, *AKT1S1* was down-regulated. We speculated that the down-regulation of *AKT1S1* may increase the activity of mTOR complex 1, thereby resulting in SONFH.

All of *BACH1*, *MGST1* and *SETD1B* were positively correlated with osteoclast differentiation. Bone remodeling is the result of a balance between osteoblast and osteoclast differentiation [43]. Bone remodeling has played a key role in the development and severity of osteonecrosis of the femoral head [44]. Nevertheless, little is known about the underlying molecular pathophysiology. Interestingly, the three key FRGs have not been investigated in SONFH to our best knowledge. Thus, we speculated that they may regulate osteoclast differentiation in SONFH.

Recently, more and more studies have suggested that infiltration of immune cell is associated with the occurrence of SONFH. For example, the activated B cells were found to have a higher frequency in SONFH patients peripheral blood compared with healthy controls [45]. Inhibitory T cells are related to the pathogenesis of nontraumatic osteonecrosis of the femoral head by regulating the bone mass balance of the femoral head [46]. In the present study, among the 28 immune cells, 20 were differential between SONFH and control groups, further suggesting the critical roles of immune cell infiltration in SONFH progression. Importantly, among these 20 immune cells, activated B cell was included and also had a higher infiltration level in SONFH group, being consistent with the report above. Mover, correlation analysis revealed that 4 key FRGs were correlated with most immune cells, suggesting that the identified key FRGs may play a role in SONFH pathogenesis through immunologic mechanisms.

Finally, we predicted the targeted drugs of the 4 FRGs. For instance, titanium dioxide had interactions with *BACH1* and *AKT1S1*. Titanium dioxide is a common component of orthopedic prosthesis [47]. Acetaminophen was predicted to have interactions with *BACH1* and *MGST1.* Acetaminophen has a central analgesic effect and has been used in osteoarthritis [48]. Investigating the interaction between key gene and targeted drug may help the development of medicine for SONFH.

## Conclusions

Four key FRGs, *AKT1S1*, *BACH1*, *MGST1* and *SETD1B*, were identified to play a critical role in the progression of SONFH through osteoclast differentiation and immunologic mechanisms. Additionally, all the 4 genes had good disease prediction effect and may act as biomarkers of the diagnosis and treatment of SONFH.

## Supplementary Information

**Additional file 1**. Pathway enrichment analysis for AKT1S1, BACH1, MGST1 and SETD1B.

## Data Availability

The datasets used and/or analyzed during the current study are available from the corresponding author on reasonable request.

## Abbreviations

DEGs: Differentially expressed genes
SONFH: Steroid-induced osteonecrosis of the femoral head
WGCNA: Weighted gene co-expression network analysis
FRG: Ferroptosis-related gene
GO: Gene ontology
KEGG: Kyoto Encyclopedia of Genes and Genomes
SVM: Support vector machine
RFE: Recursive feature elimination
